# Automated extraction protocol for quantification of SARS-Coronavirus RNA in serum: an evaluation study

**DOI:** 10.1186/1471-2334-6-20

**Published:** 2006-02-09

**Authors:** Rossa WK Chiu, Yongjie Jin, Grace TY Chung, Wing-bong Lui, Anthony TC Chan, Wilina Lim, YM Dennis Lo

**Affiliations:** 1 Centre for Emerging Infectious Diseases, The Chinese University of Hong Kong, Prince of Wales Hospital, Hong Kong; 2 Department of Chemical Pathology, The Chinese University of Hong Kong, Prince of Wales Hospital, Hong Kong; 3 Department of Clinical Oncology, The Chinese University of Hong Kong, Prince of Wales Hospital, Hong Kong; 4 Department of Health, Centre for Health Protection, Government of the Hong Kong Special Administrative Region, Hong Kong

## Abstract

**Background:**

We have previously developed a test for the diagnosis and prognostic assessment of the severe acute respiratory syndrome (SARS) based on the detection of the SARS-coronavirus RNA in serum by real-time quantitative reverse transcriptase polymerase chain reaction (RT-PCR). In this study, we evaluated the feasibility of automating the serum RNA extraction procedure in order to increase the throughput of the assay.

**Methods:**

An automated nucleic acid extraction platform using the MagNA Pure LC instrument (Roche Diagnostics) was evaluated. We developed a modified protocol in compliance with the recommended biosafety guidelines from the World Health Organization based on the use of the MagNA Pure total nucleic acid large volume isolation kit for the extraction of SARS-coronavirus RNA. The modified protocol was compared with a column-based extraction kit (QIAamp viral RNA mini kit, Qiagen) for quantitative performance, analytical sensitivity and precision.

**Results:**

The newly developed automated protocol was shown to be free from carry-over contamination and have comparable performance with other standard protocols and kits designed for the MagNA Pure LC instrument. However, the automated method was found to be less sensitive, less precise and led to consistently lower serum SARS-coronavirus concentrations when compared with the column-based extraction method.

**Conclusion:**

As the diagnostic efficiency and prognostic value of the serum SARS-CoV RNA RT-PCR test is critically associated with the analytical sensitivity and quantitative performance contributed both by the RNA extraction and RT-PCR components of the test, we recommend the use of the column-based manual RNA extraction method.

## Background

The severe acute respiratory syndrome (SARS), etiologically related to a newly emerged coronavirus (SARS-CoV) [1], caused an epidemic in 2003 with reported cases in 29 countries around the world [2]. A factor that is important in the effective control of an epidemic involves the early identification and isolation of infected individuals [3]. We have previously reported the development of a diagnostic test based on the detection of the SARS-CoV RNA in serum/plasma by real-time quantitative reverse transcriptase polymerase chain reaction (RT-PCR) [4,5]. Eighty percent of infected individuals were shown to be positive by the test on the first day of hospital admission with no false-positive results [4,5]. The serum SARS-CoV RNA concentration detected upon admission has also been shown to be predictive of the requirement for subsequent intensive care [4]. The approach has been demonstrated to be useful for serial monitoring of treatment efficacy [6].

The analytical protocol that had been developed involved the use of a manual RNA extraction method. As the demand for diagnostic testing at times of infectious disease outbreaks would be high, strategies that may enhance the throughput of analytical procedures would be advantageous. In this study, we assessed the feasibility of automating the RNA extraction procedure of the serum SARS-CoV RNA test by evaluating the performance of an automated extraction system.

## Methods

The aim of the study was to compare the efficacy of SARS-CoV RNA extraction based on the use of the QIAamp viral RNA mini kit (Qiagen, Hilden, Germany) with protocols adapted for the MagNA Pure LC instrument (Roche Diagnostics, Basel, Switzerland). The former protocol is a manual column-based method based on silica-adsorption. On the other hand, the MagNA Pure LC instrument extracts nucleic acids from biological specimens based on magnetic bead separation. The principle and general setup of the instrument had been previously described [7]. The main objective of this study was to compare the resultant analytical sensitivity and quantitative performance of the serum SARS-CoV RNA test when either the manual or automated extraction protocol was used.

### Development of an automated protocol for SARS-CoV RNA extraction

According to the manufacturer's information, 2 kits, namely the MagNA Pure LC total nucleic acid isolation kit (Roche Diagnostics) and the MagNA Pure total nucleic acid large volume isolation kit (Roche Diagnostics), are recommended for use with the MagNA Pure LC instrument for the extraction of viral DNA or RNA from serum or plasma. The main differences between the two kits lie in the starting sample volume and whether an external lysis protocol is available. The former kit processes 200 μL of serum and is compatible with the use of an external lysis protocol preinstalled in the accompanying software (MagNA Pure LC Software v.3.0, Roche Applied Science) of the MagNA Pure LC instrument. The latter kit, however, processes 1000 μL of serum and no external lysis protocol had been predefined. External lysis is a processing step whereby lysis buffer can be added to clinical specimens manually prior to the transfer of the sample and buffer mixture to the automated instrument for further downstream processing. External lysis is a desirable step for the processing of potentially infectious specimens whereby the specimens could be processed according to the recommended biosafety precautions until the pathogens are lysed and the specimen rendered safe for further processing by the MagNA Pure LC instrument.

However, the sensitive detection of SARS-CoV RNA from serum may be dependent on a large starting volume of serum. Therefore, we evaluated an in-house modification of the manufacturer's protocol for the "large volume" kit with the addition of an external lysis step. To minimise the infectious risk to the laboratory personnel, all analyses were performed using aliquots of a SARS-CoV culture isolate that had been inactivated by procedures previously described [8]. Inactivated SARS-CoV was spiked into transport medium so that the resultant mixture contained 10^8 ^copies/mL of the virus. Viral RNA was extracted from aliquots of this mixture in triplicate both according to the standard as well as modified protocols for the large volume kit. For the standard protocol, laboratory personnel were only involved with the initial transfer of 1000 μL of each specimen to individual sample cartridges placed inside the instrument after which proteinase K and buffers, including 450 μL of lysis buffer, were added by the instrument in a sequential and automated fashion. For the modified protocol, 310 μL of lysis buffer was first added to 690 μL of specimen in a biosafety cabinet to make up a final volume of 1000 μL. After vortexing, the mixture was transferred in ice to the sample cartridges on the MagNA Pure LC instrument. The instrument was then activated to run as per the standard protocol. The externally-added lysis buffer (310 μL) when mixed with the volume of lysis buffer pre-loaded on the MagNA Pure LC instrument (450 μL) amounts to a total of 860 μL of lysis buffer for 690 μL of specimen and thus contributes to a lysis buffer:sample ratio which largely resembles that adopted in the external lysis protocol of the total nucleic acid isolation kit. All viral RNA preparations extracted in this study were quantified using a real time quantitative one-step RT-PCR assay targeting the *Nucleocapsid*-gene of the SARS-CoV as previously described [4]. Briefly, the assay involves the use of the EZ r*Tth *RNA PCR reagent set (Applied Biosystems, Foster City, California) on an Applied Biosystems 7700 Sequence Detector. 12 μL of extracted viral RNA was used for amplification in a reaction volume of 25 μL.

The modified large volume protocol with the external lysis step was further compared with the external lysis protocol of the total nucleic acid isolation kit using a transport medium mixture containing 10^6 ^copies/mL of inactivated SARS-CoV. Quadruplicate extractions were performed. We also assessed the analytical sensitivities of both protocols by comparing the detection rates for aliquots of transport medium diluted to contain 10, 10^2 ^and 10^3 ^copies/mL of inactivated SARS-CoV, respectively. Five extractions were performed for each concentration. We then addressed the possibility of carry-over contamination within the MagNA Pure LC instrument by introducing nine aliquots of transport medium containing inactivated SARS-CoV ranging from 10^2 ^to 10^7 ^copies/mL alternating with aliquots of plain transport medium on the instrument and using the modified large volume protocol for extraction.

### Comparison of the automated and manual protocols for SARS-CoV RNA detection in serum

The modified large volume protocol was selected for further comparison with the performance of the column-based manual method. The manual extraction method was performed according to the manufacturer's instructions. Transport medium and pooled sera were mixed with serially diluted aliquots of inactivated SARS-CoV to produce samples containing SARS-CoV with concentrations ranging from 10^3 ^to 10^9 ^copies/mL. The pooled sera were first confirmed to be negative for SARS-CoV by the quantitative RT-PCR assay. Viral RNA was extracted from the serial samples by both the automated and manual methods and SARS-CoV RNA concentrations were determined by the quantitative RT-PCR test [4]. Viral RNA concentrations were compared using Passing-Bablok regression [9].

We next compared the effects of the RNA extraction methods on the overall assay sensitivity. Pooled sera spiked with 10, 10^2 ^and 10^3 ^copies/mL of inactivated SARS-CoV were extracted by both methods. Ten replicate extractions were performed for each concentration. The detection rate at each concentration was compared amongst the two extraction methods. Lastly, we assessed the reproducibility of both protocols by performing replicate analyses (n = 10) of two pooled sera containing 10^3 ^and 10^5 ^copies/mL of inactivated SARS-CoV, respectively. The mean and coefficient of variation were determined and compared.

### Statistical analysis

Statistical analysis was performed using the MedCalc software version 8.0.

## Results

### Evaluation of the modified protocol of the MagNA Pure LC total nucleic acid large volume kit and comparisons with the MagNA Pure LC total nucleic acidisolated kit

Triplicate viral RNA extractions of the transport medium mixture containing 10^8 ^copies/mL inactivated SARS-CoV by the standard protocol of the large volume kit yielded SARS-CoV RNA concentrations of 3.11 × 10^8^, 3.14 × 10^8 ^and 3.17 × 10^8 ^copies/mL. By incorporating an external lysis procedural step, the modified protocol yielded 4.92 × 10^8^, 5.20 × 10^8 ^and 5.48 × 10^8 ^copies/mL. As the modified large volume protocol yielded results comparable to that of the standard large volume protocol, the former was used for further comparison with the external lysis protocol of the "total nucleic acid" kit which uses a starting sample volume of 200 μL. Quadruplicate analysis of the transport medium mixture containing 10^6 ^copies/mL SARS-CoV yielded 6.57 × 10^6^, 7.41 × 10^6^, 1.34 × 10^7 ^and 6.17 × 10^7 ^copies/mL when the large volume kit was used. When the total nucleic acid kit was used, the results were 8.20 × 10^6^, 8.40 × 10^6^, 1.05 × 10^7 ^and 6.80 × 10^7 ^copies/mL.

The analytical sensitivities contributed by the two kits were next compared. Viral RNA extracted by both kits was detectable in all five replicates when the sample contained 10^3 ^copies/mL SARS-CoV. However, when the sample contained 10^2 ^copies/mL SARS-CoV, the large volume kit yielded positive detection in all five replicates, while SARS-CoV was only detected from three replicates when extracted by the total nucleic acid kit. Furthermore, when the sample contained 10 copies/mL SARS-CoV, the large volume kit yielded positive detection in four replicates, while viral RNA extractions from the total nucleic acid kit was only positive in one replicate. These data suggest that the modified protocol of the large volume kit has comparable performance with the total nucleic acid kit for the extraction of samples containing high SARS-CoV concentration, but enabled more sensitive detection when samples containing low levels of SARS-CoV were extracted. By aligning samples positive and negative for SARS-CoV in an alternating manner for extraction by the modified protocol of the large volume kit, there was no evidence of carry-over contamination. All the negative samples were indeed tested negative regardless of the magnitude of the SARS-CoV concentrations (ranging from 10^2 ^to 10^7 ^copies/mL) in the adjacent wells. Thus, the modified protocol of the large volume kit was selected for further evaluation for SARS-CoV RNA extraction from serum.

### SARS-CoV RNA detection in serum extracted by the automated and manual methods

**Figure 1:**
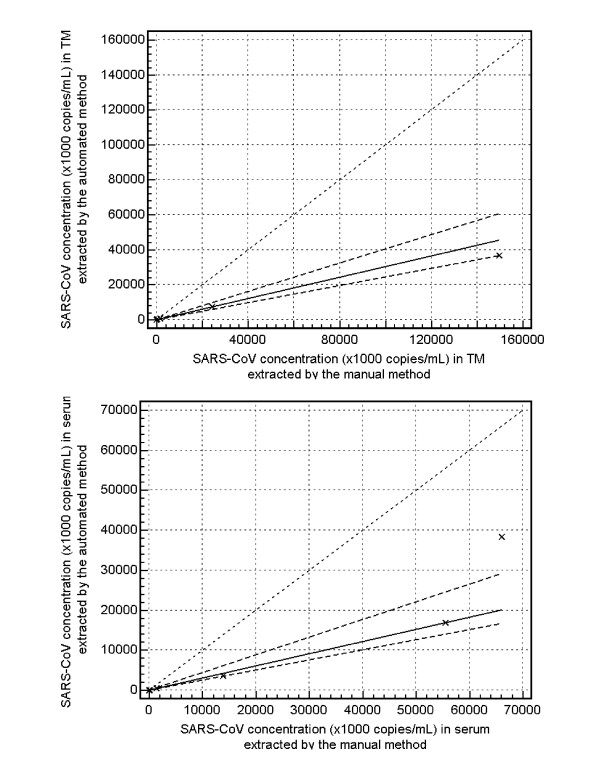
**Passing-Bablok regression analysis of SARS-CoV RNA concentrations in (A) transport medium and (B) serum extracted by the automated and manual methods**. The regression line is indicated by the solid line, with the confidence intervals marked as dashed lines. The identity line (x = y) is indicated as the dotted line. TM: transport medium.

Serially diluted inactivated SARS-CoV isolate in transport medium was extracted by both the column-based manual method and the MagNA Pure LC instrument using the modified large volume protocol with external lysis. The SARS-CoV concentrations from both series of viral RNA extracts were compared using the Passing-Bablok regression method [9]. The Passing-Bablok procedure is a linear regression method developed for method comparison evaluations without dependence on the assignment of either one of the two compared methods as the reference. Furthermore, it makes no assumption on the distribution of sample data and measurement errors of the compared methods. The Passing-Bablok comparison of the SARS-CoV concentrations in transport medium as extracted by the two methods is presented in Figure 1A. The quantitative relationship can be described by y = -207.0 + 0.305x (95% confidence intervals for the slope, 0.260 to 0.407; and y-intercept, -661.7 to 128.6). The slope of the regression line being less than 1 suggests there is a proportional negative bias in SARS-CoV concentration extracted by the automated method when compared with the manual method.

A similar comparison was performed for serially diluted SARS-CoV mixture in pooled sera. Figure 1B illustrates the Passing-Bablok comparison. The quantitative relationship can be described by y = -62.4 + 0.304x (95% confidence intervals for the slope, 0.252 to 0.442; and y-intercept, -205.6 to 380.4). The slope of the regression line being less than 1 also suggests the presence of a proportional negative bias in serum SARS-CoV concentration extracted by the automated method when compared with the manual method.

The effect of the automated and manual methods on the overall assay sensitivity was next compared. Results for this part of the study are summarized in Table 1. SARS-CoV RNA was detectable from all ten replicates when the serum aliquots containing 10^3 ^copies/mL SARS-CoV were extracted by either methods. For serum containing 10^2 ^copies/mL SARS-CoV, nine and seven of the replicates were tested positive when viral RNA was extracted by the manual and automated methods, respectively. For serum containing 10 copies/mL SARS-CoV, four of the replicates were positive when extracted by the manual method and only one was positive when the replicates were extracted by the automated method.

**Table 1:** Analytical sensitivity and precision comparisons of the manual and automated methods.

	**Sensitivity comparison**			**Precision comparison**					
**SARS-CoV RNA Concentration (copies/mL)**	10	10^2^	10^3^	10^3^			10^5^		
				
				mean	S.D.	CV (%)	mean	S.D.	CV (%)

**Automated**	1/10^a^	7/10	10/10	349	268	76.9	35052	17892	51
**Manual**	4/10	9/10	10/10	1539	697	45.3	83591	39606	47.4

^a^number of replicates with positive detection of SARS-CoV RNA.

S.D. denotes standard deviation and CV denotes coefficient of variation.

To assess the effects of the two extraction protocols on the precision or reproducibility of the quantitative SARS-CoV RT-PCR assay, RNA extractions by each protocol were repeated 10 times for serum aliquots containing SARS-CoV concentration well above the detection limit of the assay, namely 10^3 ^and 10^5 ^copies/mL. Results for this part of the study are summarized in Table 1. For serum SARS-CoV concentration at 10^3 ^copies/mL, the overall assay coefficient of variation (standard deviation/mean × 100%) was 45.3% and 76.9% when RNA extractions from the manual and automated methods were quantified, respectively. For serum SARS-CoV concentration at 10^5 ^copies/mL, the assay coefficient of variation was 47.4% for the manual RNA extraction and 51.0% when automated RNA extraction was used.

## Discussion

In an attempt to increase the throughput of a previously developed quantitative serum SARS-CoV RNA RT-PCR assay [4,5], we evaluated the feasibility of automating the RNA extraction procedure through the use of the MagNA Pure LC instrument (Roche Diagnostics). Reagent kits suitable for the extraction of viral RNA from serum and plasma as recommended by the instrument manufacturer were evaluated. As the extraction procedure should conform to the biosafety practices recommended by the World Health Organization [10], a modified protocol which incorporates an external lysis processing step for the MagNA Pure LC total nucleic acid large volume kit (Roche Diagnostics) was developed. The World Health Organization recommends that nucleic acid extraction procedures for SARS-CoV involving untreated specimens should first be performed under biosafety level 2 facilities with additional level 3 work practices [10]. After the viral particles had been lysed or inactivated, the specimens could be handled according to standard level 2 practices. We showed that the use of the large volume kit resulted in better analytical sensitivity when compared with the total nucleic acid kit as evident by the higher rates of positive detection among samples containing low concentrations of SARS-CoV. Furthermore, the MagNA Pure LC system was shown to be free from problems of carry-over contamination.

The automated extraction method involving the use of the large volume kit with the external lysis procedure was further compared with the quantitative performance of a previously described manual viral RNA extraction method based on the use of the QIAamp viral RNA mini kit (Qiagen). Viral RNA extracted from the automated method led to SARS-CoV concentrations that were consistently lower than that extracted by the manual method across a wide range of SARS-CoV concentrations in both transport medium and serum. Furthermore, better detection rates were observed for serum containing low concentrations of SARS-CoV when extracted by the manual method in comparison with the automated method. The manual method also contributed to better overall analytical precision as evident by the lower coefficients of variation.

## Conclusion

We have developed a modified protocol based on the use of the MagNA Pure LC large volume kit (Roche Diagnostics) which is more sensitive than the predefined external lysis protocol of the MagNA Pure LC total nucleic acid kit (Roche Diagnostics). Albeit the convenience and potential improvement in throughput offered by an automated protocol, our evaluation revealed that the automated viral RNA extraction protocol is less sensitive, less precise and produced quantitative results that were consistently lower than those of the column-based manual extraction method. Though the reasons for the observed differences in kit performance is not known at present, we recommend the use of the column-based manual RNA extraction method as the diagnostic performance of the serum SARS-CoV RNA quantitative RT-PCR test [4,5] is critically associated with the analytical sensitivity contributed both by the RNA extraction and RT-PCR components of the test,. Furthermore, as it has been previously shown that the serum SARS-CoV concentration has prognostic implications [4] and serial assessment is useful for the monitoring of patient progress [5,6], the superior quantitative performance and precision of the column-based extraction method are additional features that favour its use over the automated protocol.

## List of abbreviations

SARS, severe acute respiratory syndrome; SARS-CoV, SARS-coronavirus, RT-PCR, reverse transcriptase polymerase chain reaction.

## Competing interests

Patent applications covering aspects of the serum SARS-CoV RNA quantitative RT-PCR test have been filed by The Chinese University of Hong Kong.

## Authors' contributions

RWKC and YMDL designed the study. RWKC interpreted the data and drafted the manuscript. YJ, GTYC and WBL performed the molecular and data analyses. WL provided the inactivated viral material. ATCC provided the expertise for the use of the MagNA Pure LC instrument. All authors read and approved the final manuscript.

## Pre-publication history

The pre-publication history for this paper can be accessed here:



## References

[B1] Peiris JS, Yuen KY, Osterhaus AD, Stohr K (2003). The severe acute respiratory syndrome. N Engl J Med.

[B2] WHO Summary of probable SARS cases with onset of illness from 1 November 2002 to 31 July 2003. http://www.who.int/csr/sars/country/table2004_04_21/en/.

[B3] Lee N, Hui D, Wu A, Chan P, Cameron P, Joynt GM, Ahuja A, Yung MY, Leung CB, To KF, Lui SF, Szeto CC, Chung S, Sung JJ (2003). A major outbreak of severe acute respiratory syndrome in Hong Kong. N Engl J Med.

[B4] Ng EKO, Hui DS, Chan KCA, Hung ECW, Chiu RWK, Lee N, Wu A, Chim SSC, Tong YK, Sung JJ, Tam JS, Lo YMD (2003). Quantitative analysis and prognostic implication of SARS coronavirus RNA in the plasma and serum of patients with severe acute respiratory syndrome. Clin Chem.

[B5] Ng EKO, Ng PC, Hon KL, Cheng WT, Hung ECW, Chan KCA, Chiu RWK, Li AM, Poon LLM, Hui DS, Tam JS, Fok TF, Lo YMD (2003). Serial analysis of the plasma concentration of SARS coronavirus RNA in pediatric patients with severe acute respiratory syndrome. Clin Chem.

[B6] Lee N, Chan KCA, Hui DSC, Ng EKO, Wu A, Chiu RWK, Wong VW, Chan PK, Wong KT, Wong E, Cockram CS, Tam JS, Sung JJS, Lo YMD (2004). Effects of early corticosteroid treatment on plasma SARS-associated Coronavirus RNA concentrations in adult patients. J Clin Virol.

[B7] Kessler HH, Muhlbauer G, Stelzl E, Daghofer E, Santner BI, Marth E (2001). Fully automated nucleic acid extraction: MagNA Pure LC. Clin Chem.

[B8] Drosten C, Doerr HW, Lim W, Stohr K, Niedrig M (2004). SARS molecular detection external quality assurance. Emerg Infect Dis.

[B9] Passing H, Bablok W (1983). A new biometrical procedure for testing the equality of measurements from two different analytical methods. Application of linear regression procedures for method comparison studies in clinical chemistry, Part I. J Clin Chem Clin Biochem.

[B10] WHO WHO post-outbreak biosafety guidelines for handling of SARS-CoV specimens and cultures. http://www.who.int/csr/sars/biosafety2003_12_18/en/.

